# Convergence of evidence from a methylome-wide CpG-SNP association study and GWAS of major depressive disorder

**DOI:** 10.1038/s41398-018-0205-8

**Published:** 2018-08-22

**Authors:** Karolina A. Aberg, Andrey A. Shabalin, Robin F. Chan, Min Zhao, Gaurav Kumar, Gerard van Grootheest, Shaunna L. Clark, Lin Y. Xie, Yuri Milaneschi, Brenda W. J. H. Penninx, Edwin J. C. G. van den Oord

**Affiliations:** 1 Center for Biomarker Research and Precision Medicine, Virginia Commonwealth University, Richmond, VA USA; 2 Department of Psychiatry, Amsterdam Neuroscience, VU University Medical Center/GGZ inGeest, Amsterdam, The Netherlands

## Abstract

DNA methylation is an epigenetic modification that provides stability and diversity to the cellular phenotype. It is influenced by both genetic sequence variation and environmental factors, and can therefore potentially account for variation of heritable phenotypes and disorders. Therefore, methylome-wide association studies (MWAS) are promising complements to genome-wide association studies (GWAS) of genetic variants. Of particular interest are methylation sites (CpGs) that are created or destroyed by the alleles of single-nucleotide polymorphisms (SNPs), as these so-called CpG-SNPs may show variation in methylation levels on top of what can be explained by the sequence variation. Using sequencing-based data from 1132 major depressive disorder (MDD) cases and controls, we performed a MWAS of 970,414 common CpG-SNPs. The analysis identified 27 suggestively significant (*P* < 1.00 × 10^−5^) CpG-SNPs associations. Furthermore, the MWAS results were over-represented (odds ratios ranging 1.36–5.00; *P* ranging 4.9 × 10^−3^–8.1 × 10^−2^) among findings from three recent GWAS for MDD-related phenotypes. Overlapping loci included, e.g., *ROBO2*, *ASIC2*, and *DCC*. As the CpG-SNP analysis accounts for the number of alleles that creates CpGs, the methylation differences could not be explained by differences in allele frequencies. Thus, the results show that the MWAS and GWASs provide independent lines of evidence for the involvement of these loci in MDD. In conclusion, our methylation study of MDD contributes novel information about loci of relevance that complements previous findings and generates new hypothesis about MDD etiology, such as that the functional effects of genetic association may be partly mediated and/or enhanced by the methylation status in these loci.

## Introduction

Major depressive disorder (MDD) is a complex disorder that is characterized by persistent dysphoria and is often accompanied by considerable morbidity^1–3^ and mortality^2,4^. Because MDD has a lifetime prevalence of almost 15%^5^, tends to start early in life^6^, and is often chronic^7,8^, it is the leading contributor to disability worldwide^9,10^. In comparison with other (psychiatric) disorders, discerning the biological basis of MDD has been difficult. Only very recently, a number of genetic variants were identified and replicated^11,12^. However, these variants had small effect sizes and explained only a small proportion of the disease risk.

DNA methylation is an epigenetic modification that provides stability and diversity to the cellular phenotype. Because methylation is dynamic in nature and can be altered by environmental factors, it can potentially account for key clinical features of MDD such as its episodic nature or mediate the effects of environmental risk factors such as stress^13–15^. Therefore, methylome-wide association studies (MWAS), which test a genome-wide set of methylation sites for association with an outcome of interest, are promising complements to genome-wide association studies (GWAS) of genetic variants. Of particular interest are methylation sites (CpGs) that are created or destroyed by single-nucleotide polymorphisms (SNPs). These sites, commonly referred to as CpG-SNPs, may show variation in both methylation and sequence, and may therefore convey information beyond either of the two data types alone. However, methylation-dependent association signals in CpG-SNPs are not captured by GWAS and are very poorly captured by a regular MWAS. Therefore, a specific CpG-SNP analysis is needed to detect these signals.

Regular GWAS studies detect differences in allele frequencies between cases and controls. In contrast, a CpG-SNP analysis tests whether groups of cases and controls with the same genotype show differences in methylation at these sites. Thus, these two analyses capture different signals. Similarly, while a regular MWAS detects differences in methylation it does not account for differences in genotype and will therefore often lack the statistical power to detect association signals for CpG-SNPs. A CpG-SNP MWAS remedies this by including information on the actual genotypes of each subject.

Furthermore, the link between sequence variation and methylation levels at these sites may allow CpG-SNPs to function as important cis-regulatory polymorphisms that connect genetic variation to variation in methylation. For example, the alleles present and the methylation levels observed at a specific CpG-SNP have been associated with a variety of regulatory functions^16–19^. In addition, in a high-density analysis of methylation quantitative trait locus (meQTL), CpG-SNPs were involved in the majority of all identified meQTLs^20^.

To study whether CpG-SNPs contribute to MDD disease risk, we used a sequencing-based approach that provides nearly complete coverage of all CpGs^21,22^, including close to 1 million CpG-SNPs. To further explore the MWAS findings and their potential relevance for MDD, we also tested for their overlap with results from recent GWASs.

## Materials and methods

### Description of the NESDA sample

DNA from blood was obtained from 1200 individuals from the Netherlands Study of Depression and Anxiety (NESDA). MDD was diagnosed using the DSM-IV-based Composite International Diagnostic Interview (CIDI version 2.1) that was administered by specially trained research staff^23^. In addition, to a current MDD diagnosis, cases had a score >14 on the IDS-SR_30_^24^, a 30-item self-report measure of depression symptoms. Controls had no lifetime psychiatric disorders and an IDS-SR_30_ score <14. The sample selection was further based on good quality GWAS genotype information available from a previous investigation^25^ (for a summary description, see the Supplementary Note). For further details about NESDA, and demographic and clinical characteristics of participants used for the present study, see Table S1. The study was approved by the ethical committees of all participating locations, and participants provided written informed consent.

### Assaying the methylome with MBD-Seq

We assayed the methylome using an optimized protocol for methyl-CG binding domain sequencing (MBD-Seq) that provides almost complete coverage of all CpGs in the genome^21^. In short, we used ultrasonication to shear genomic DNA into, on average, 150 bp fragments followed by enrichment with MethylMiner^™^ (Invitrogen) to capture the methylated fraction of the genome. The captured fragments were eluted and used to create a barcoded sequencing library for each methylation capture. Labeled sequencing fragment libraries were pooled in equal molarities and sequenced on a NextSeq500 instrument (Illumina). To ensure consistency in the sample preparation, MethylMiner captures and library constructions were both performed using Biomek NxP robotics (Beckman Coulter). Samples were performed in a randomized order and all labtecnical procedures were performed blind to any phenotype information. The sequence reads were aligned to the human reference genome (hg19/GRCh37) using Bowtie2^26^.

### Data processing and quality control

Quality control and data processing (Supplementary Note) were performed using our RaMWAS Bioconductor package, which is specifically designed for large-scale methylation studies. After rigorous quality control of samples, reads, and CpGs, 1132 subjects (320 controls and 812 cases) with an average of 48.7 million reads per sample (=81.9% of all reads) remained. For each of these individuals, our dataset included commonly methylated high-quality methylation information for 21,869,561 CpGs^27^. Among these, 970,414 were common CpG-SNPs (CpGs created/destroyed by SNPs with minor allele frequency > 10%) that were used for MWAS. To identify the CpG-SNPs we used directly genotyped and imputed genotype information (Supplement) from the NESDA participants. The imputed SNPs were filtered by imputation R2 ≥ 0.9 and minor allele frequency ≥ 0.1 in cases and controls. Finally, an in silico experiment described elsewhere^28^ was used to remove CpG-SNPs in loci showing alignment problems.

### MWAS of CpG-SNPs

To test for association between the methylation level at each CpG-SNP and MDD, we performed a regression analysis with four sets of covariates. First, we regressed out 19 assay-related variables (i.e., potential technical artifacts) including the quantity of methylation-enriched DNA captured, sample batches, and peak location^22^. Second, we regressed out the demographic variables age and sex. Third, to avoid confounding due to cell-type heterogeneity, we regressed out blood cell type proportions as estimated by the methylation data^29^ using MBD-Seq “reference methylomes” we generated after isolating all common cell types in blood^30^. Fourth, principle component analysis was used to capture any remaining unmeasured source of variation. Specifically, using a scree test we selected the first principle component.

**Fig. 1:**
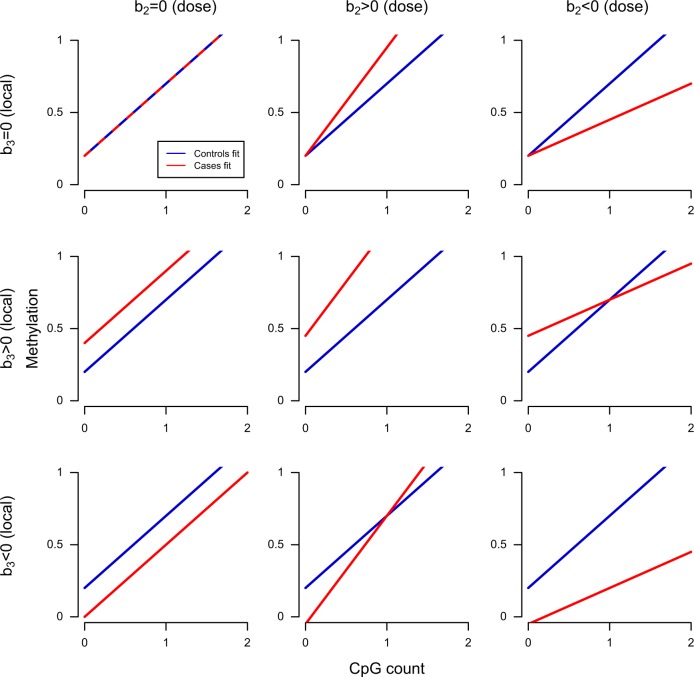
Overview of possible scenarios for the regression lines. Keeping the *b*_0_ and *b*_1_ parameters constant while altering the *b*_2_ and *b*_3_ parameters result in nine possible scenarios for the regression lines. The *b*_0_ parameter is kept at 0.2 and *b*_1_ (the meQTL effect) is equal to 1 for all plots. The value for the *b*_2_ parameter (the dose effect) was altered between a value equal to zero (0; left column), a positive value (0.5; middle column), and a negative value (−0.5; right column). Similarly, the value for the *b*_3_ parameter (the local effect) was altered between a value equal to zero (0; top row), a positive value (0.25; middle row), and a negative value (−0.25; bottom row). A non-zero value for *b*_3_ means that the locus is affected by a case-control differences from nearby CpGs (i.e., a “local effect”). This effects is independent of the effect from *b*_2_ and can either enhance or (partly) diminish the dose effect

The MWAS was performed by fitting the following regression equation:^31^

$$Y = b_0 + b_1{\mathrm{CpG}} - {\mathrm{SNP}} + b_2\left( {{\mathrm{CpG}} - {\mathrm{SNP}} \times {\mathrm{MDD}}} \right) + b_3{\mathrm{MDD}} + b_4X_1 + \ldots + b_kX_k + E,$$

where *Y* are the CpG scores, *b*_0_ is the intercept of the regression line, *b*_4_…*b*_k_ the effects of covariates, and *E* are the residual effects. The CpG-SNP is coded as 0, 1, and 2, which corresponds to having 0, 1, or 2 copies of the SNP allele that creates/destroys the CpG relative to the reference genome. MDD is coded 0 for controls and 1 for cases. Figure 1 describes nine scenarios for how the regression lines change with alterations of the *b*_2_ and *b*_3_ parameters, when *b*_1_ is equal to 1. A non-zero value of parameter *b*_1_ indicates that the site is methylated with the amount of methylation being proportional to the number of CpGs (i.e., has a methylation quantitative locus or “meQTL effect”). Parameter *b*_2_ estimates the case-control difference at the CpG-SNP site that is proportional to the number of CpGs (i.e., the “CpG-SNP dose effect”). Parameter *b*_3_ captures case-control differences from nearby sites and thus do not depend on the number of CpG creating alleles of the SNP (i.e., a “local effect”). MBD-Seq assays the methylation of regions that are about the size of the sequenced fragments (~150 bp). Therefore, part of the differences observed at the CpG-SNP may reflect the effects of nearby CpGs resulting in non-zero values *b*_3_. In the overall association (i.e., “CpG-SNP MWAS”) we tested the null-hypothesis, H_0_: *b*_2_ = *b*_3_ = 0.

### Permutation of CpG-SNP MWAS to study the null distribution

To test if the lambda observed for the MDD CpG-SNP MWAS was caused by associations to the outcome variable, or if it was caused by that the test statistic distribution did not follow the theoretical null we used permutations. Using exactly the same dataset, we performed MWAS for 100 permutations of the MDD outcome variable and recorded the lambdas. Next, the observed association *P*-values from the MDD CpG-SNP MWAS were corrected for the average permutation-obtained lambda.

### Replication of cumulative MWAS signals by resampling

To study the significance of the cumulative MWAS signals, we used the “ramwas7riskScoreCV” function in RaMWAS. Specifically, the function uses elastic nets^32–34^ as implemented in the R Glmnet package. Elastic nets are akin to multiple regression analysis but suitable for our scenario where the number of predictors is much larger than the number of observations. Elastic nets were fitted by setting the alpha parameter to zero (i.e., ridge regression that retains all predictive sites in the model). To avoid overfitting, *k*-fold cross-validation is used^35^. That is, the sample was randomly partitioned into *k* = 10 equal sized subsamples. Of the *k* subsamples, *k*−1 are used as a “training” set to fit the elastic net and obtain weights for each predictive methylation site. The estimated weights are then used in the remaining “test” set to predict the outcome from the methylation data. By alternating the subjects used in the training and test sets, predictions are obtained for all subjects in the study. RaMWAS repeats the entire cycle of CpG-SNP selection through MWAS followed by estimation of prediction weights using elastic nets for each of the *k*-folds. Because both the selection of CpG-SNPs and estimation of their weights are not affected by the participants in the test set, we obtain unbiased predictions of the outcome for each subject. Furthermore, the score of CpG-SNPs is for an important part determined by the number of CpGs. To capture only effects associated with MDD, we removed the effect of the number of CpGs from the methylation score prior to conducting the “in sample” replication. By testing whether these methylation predictions are significantly correlated with actual MDD status, we performed an “in sample” replication of the cumulative MWAS signal.

### Permutation-based enrichment test of overlap

To perform enrichment tests of the overlap between datasets we used the “shiftR” R-package. shiftR first maps the two datasets to each other based on chromosomal location. In our analyses, no flanking regions were used. Thus, for SNPs we considered a single base position and for CpGs we considered two bases. Next, the *P-*values are used to cross-classify each mapped marker in the two datasets as being in the top or bottom. Based on the resulting 2 by 2 tables as input, shiftR tests the null hypothesis that the enrichment odds ratio equals 1. To perform these test, shiftR uses circular permutations^36^. Specifically, through fast bitwise operations, it shifts the mapping of the two datasets by a single random integer in each permutation. This approach to generate the empirical test statistic distribution under the null hypothesis preserves the correlational structure of the data. We used 1 million permutations for each test. Multiple thresholds can be specified to define “top findings” (i.e., for our analyses we used the top 1 and 5%). To account for this “multiple testing”, the same thresholds are used in the permutations where the test statistic distribution under the null hypothesis is generated from the most significant (combination of) thresholds.

### Three GWAS

Three independent (meta-analysis of) GWASs were recently reported for MDD or related phenotypes. Similar to the phenotyping in the NESDA sample, the 23andMe study^12^ and the study by the Converge Consortium^37^ determined phenotype status using information about current or prior MDD diagnosis. In contrast, the GWAS meta-analysis performed by the Social Science Genetics Association Consortium (SSGAC)^11^ studied depressive symptoms, which for the majority of the individuals (>105,000 individuals out of 161,460) were assessed based on self-reported frequency an individual had experienced feelings of unenthusiasm/disinterest and depression/hopelessness during the past 2 weeks. Thus, this assessment was not a clinical diagnosis of depression nor a validated method for assessing depression symptoms. In contrast, when SSGAC studied neuroticism, an MDD-related phenotype, the status for the majority of individuals was assessed using a validated questionnaire that applied different harmonized neuroticism assessment batteries (*n* = 63,661) and a 12-item version of the Eysenck Personality Inventory Neuroticism^38^ (*n* = 107,245). Therefore, for the purpose of comparison with our MWAS for MDD, we used the SSGAC GWAS meta-analysis results of neuroticism^11^.

For calculating the enrichment test statistic, shiftR classifies markers as being among the top vs. bottom results. However, from the 23andMe study, we could only get access to the *P-*values from the top 10,000 SNPs. To address this restriction we used SNPs retained in the multiple Psychiatric Genetic Consortia (http://www.med.unc.edu/pgc) studies after quality control. After removing the 10,000 top 23andMe SNPs, we assumed that these common and QC’ed SNPs were likely tested or were in LD with tested SNPs in the 23andMe study but yielded *P*-values lower than those of the top 10,000. The top 10,000 SNPs all had *P*-values < 10^−5^. To define a second threshold for the 23andMe study, we also selected the 745 SNPs with *P*-values < 10^−8^. To account for this “multiple testing”, the same two thresholds were used in the permutations. To maximize the compatibility of the analysis, all GWAS datasets were subjected to the same procedure as used for the 23andMe study.

## Results

### Methylome-wide CpG-SNP analysis

**Fig. 2:**
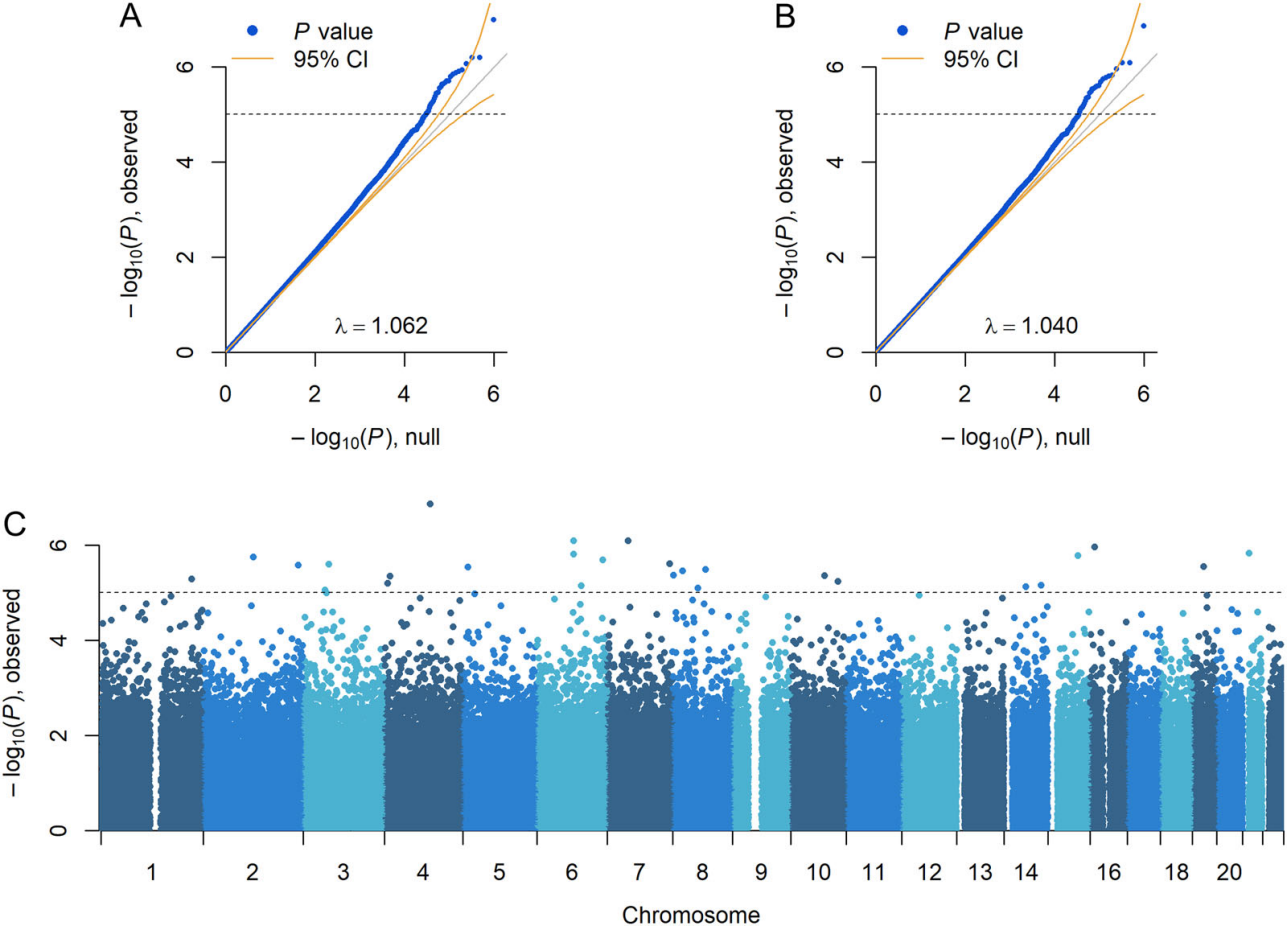
Q-Q plots and Manhattan plot of CpG-SNP MWAS. **a** Quantile-Quantile plot of the CpG-SNP MWAS before correction. The observed *P*-values, on a –log_10_ scale, are plotted against their expected values (gray main diagonal line) under the null hypothesis assuming none of the sites have an effect. Yellow lines indicate the 95% confidence intervals (CI). **b** Quantile-Quantile plot of the CpG-SNP MWAS after correction for permutation-obtained lambda. The deviation of *P-*values from the main diagonal indicates that, even after correction, there are potentially many markers associated with MDD. **c** Manhattan plot of the CpG-SNP MWAS. The plot shows the MWAS *P-*values on a –log_10_ scale (*y*-axis) by their chromosomal location (*x*-axis). The dashed line marks the threshold for suggestively significant findings (*P* = 1 × 10^−5^)

We utilized the methylation data in combination with genotype information from the same individuals to perform a MWAS involving 970,414 common CpG-SNPs. Permutations of the MWAS generated an average lambda of 1.02 with a 95% confidence interval from 1.0087 to 1.0321. Thus, as shown in the Q-Q plot (Fig. 2a), the slightly inflated lambda (lambda = 1.062) observed for the MDD CpG-SNP MWAS is likely caused by a combination of true associations and by that the test statistic distribution did not follow the theoretical null distribution. As it would be practically non-feasible (too time-consuming) to obtain permutation *P*-values for each site we instead control for the deviation in the theoretical null distribution. Thus, the *P*-values were corrected for the average permutation-obtained lambda (Fig. 2b).

The Manhattan plot (Fig. 2c) shows 27 suggestively significant loci (*P* < 1.00 × 10^−5^ after lambda correction) across the genome (Table 1). In Fig. S1, we show the regression plots for all the 27 sites. Twenty-five (92.6%) of the sites showed that the methylation levels were dependent on the number of CpG alleles (i.e., there was a significant meQTL effect) and 23 sites (85.2%) showed that this effect was different between cases and controls (i.e., there was a significant CpG-SNP dose effect). Thus, the associations observed for the two sites lacking CpG-SNP dose effects, as well as for the two sites that did not show significant meQTL effects, are likely caused by local effects from nearby CpGs.

**Table 1:** CpG-SNP MWAS findings with *P* < 1.00e-5

Chr.	Position (bp)	Gene	meQTL effect		CpG-SNP dose effect		Local effect		CpG-SNP MWAS
			Beta	*P*-value	Beta	*P*-value	Beta	*P*-value	Corrected *P-*value
4*	111,642,419		0.7905	5.38E-17	−0.5752	2.17E-07	0.0131	8.22E-01	1.37E-07
6*^R^	89,399,125	*RNGTT*	0.5802	6.54E-12	−0.4285	1.15E-05	0.0439	3.75E-01	8.11E-07
7*^R^	50,638,462		0.1557	3.21E-02	−0.3998	4.50E-06	0.0005	9.93E-01	8.14E-07
16*	11,045,718	*CLEC16A*	0.5358	2.39E-12	−0.201	2.29E-02	−0.1615	3.28E-03	1.09E-06
21	15,628,668	*ABCC13*	0.5026	2.10E-10	0.4714	7.79E-07	−0.0467	3.95E-01	1.48E-06
6*^R^	89,399,133	*RNGTT*	0.5226	3.51E-10	−0.4055	2.61E-05	0.0489	3.18E-01	1.56E-06
15	73,204,661		0.5514	6.76E-20	0.2885	3.53E-05	0.017	7.15E-01	1.68E-06
2	121,561,733	*GLI2*	0.3263	1.27E-08	0.277	3.59E-05	−0.0625	3.64E-01	1.80E-06
6*	160,652,677	*SLC22A2*	1.2566	2.99E-70	−0.3981	4.86E-07	0.057	1.79E-01	2.03E-06
7* ^R^	152,831,050		0.8992	1.91E-46	−0.2612	2.25E-04	−0.3831	3.00E-07	2.47E-06
3*	62,786,520	*CADPS*	1.3587	1.30E-80	−0.3623	2.48E-06	0.0072	8.45E-01	2.51E-06
2	231,351,298	*SP100*	0.7877	8.99E-46	0.2316	2.33E-04	0.0265	5.81E-01	2.63E-06
19*	28,066,083		1.0848	7.70E-90	−0.1779	2.38E-03	−0.0676	1.18E-01	2.84E-06
5	12,652,832	*CT49*	0.7771	4.22E-49	0.2577	2.53E-05	0.0061	8.82E-01	2.88E-06
8*	80,627,057		1.0881	2.56E-104	−0.1471	5.42E-03	−0.0544	2.58E-01	3.27E-06
8*	25,147,472	*DOCK5*	1.1061	5.52E-58	−0.2184	4.19E-03	−0.0949	6.20E-02	3.48E-06
8*	3,190,003	*CSMD1*	0.4985	2.96E-16	−0.1445	4.05E-02	−0.1214	7.17E-02	4.28E-06
10	83,266,584		0.0338	5.95E-01	−0.3749	5.65E-07	0.3099	7.66E-05	4.37E-06
4	13,762,888		0.0642	2.36E-01	0.0668	2.95E-01	−0.212	4.16E-06	4.49E-06
1* ^R^	221,200,361		0.7162	5.21E-27	−0.2521	1.15E-03	−0.2787	7.41E-07	5.15E-06
10* ^R^	116,028,591	*VWA2*	0.2869	3.87E-05	−0.2984	2.68E-04	−0.239	1.81E-06	5.75E-06
4 ^R^	8,327,194		0.5927	2.07E-16	0.0094	9.10E-01	0.2675	1.12E-04	6.41E-06
14	90,682,467		0.8614	6.29E-66	−0.0534	3.30E-01	−0.1368	1.96E-02	6.94E-06
6* ^R^	107,694,468	*PDSS2*	0.4479	9.31E-09	−0.4037	7.64E-06	−0.0267	6.10E-01	7.20E-06
14* ^R^	53,232,347	*STYX*	0.9249	6.43E-75	−0.2267	3.52E-05	−0.3222	1.00E-06	7.49E-06
8* ^R^	61,460,116	*RAB2A*	0.1289	4.16E-02	−0.3582	1.56E-06	−0.1248	2.28E-03	8.09E-06
3*	54,329,568	*CACNA2D3*	0.7707	3.49E-43	−0.2019	1.52E-03	−0.0461	3.62E-01	8.74E-06

The overall association tests both the CpG-SNP dose effect (*b*_2_) and local effect or H_0_: *b*_2_ = *b*_3_ = 0, meQTL tests H_0_: *b*_1_ = 0, CpG-SNP dose effect tests H_0_: *b*_2_ = 0, and local effect tests H_0_: *b*_3_ = 0. All data corresponds to the effect of the CpG creating allele. ^R^ indicates that the SNP destroys the CpG allele in the reference genome, and thus the data was converted to reflect the effect of the CpG creating allele. Asterisk indicates sites showing a distinct pattern where methylation increases with the number of CpG alleles present (positive meQTL effect) but where this increase was attenuated in cases compared to controls (negative CpG-SNP dose effect)

*Chr.* chromosome

Focusing on the 23 CpG-SNPs with both meQTL and CpG-SNP dose effects, we identified five sites (21.7%) with a positive dose effect. These sites showed a consistent pattern where the case-control difference gets bigger with more CpG-creating alleles but where the cases show higher methylation levels than the controls. The reaming 18 sites (78.3%) showed a negative dose effect. Thus, the negative dose effect occurred significantly more often (*P* = 0.0040) than expected by chance. A negative dose effect translates to (Fig. 1, right column) a case-control difference that gets bigger with more CpG-creating alleles and where the cases show lower methylation levels than the controls. As was shown in Fig. 1, the CpG-SNP MWAS associations (detected with MBD-Seq data) is in addition to a meQTL effect and a dose effect, also influenced by the local effect from nearby CpGs. This local effect can both enhance or diminish the CpG-SNP dose effect.

The deviation of the observed *P-*values from the main diagonal, observed in the Q-Q plot (Fig. 2b) after correction for artificial inflation, suggests multiple sites are potentially associated with MDD. To study the significance of the cumulative CpG-SNP MWAS signal for large portions of the top markers, we used a resampling approach that fits elastic nets and employs *k*-fold cross-validation to avoid overfitting and obtain an unbiased estimate of the cumulative effect across markers. Results showed that the cumulative association was significant (*P* = 4.01 × 10^−8^), with the signal coming from the top 15,000 markers.

### Overlap between CpG-SNP MWAS and GWAS

When comparing our MWAS results with three recent GWAS, a significant, or trend toward, enrichment was observed for all three GWASs when using the top 1% of results (the 1% threshold) for the CpG-SNP MWAS and the most stringent threshold for each of the three GWAS (Table 2). The highest enrichment was observed with the 23andMe study (*P* = 4.9 × 10^−3^, OR = 5.00) followed by SSGAC (*P* = 3.8 × 10^−2^, OR = 1.42) and Converge (*P* = 8.1 × 10^−2^, OR = 1.36). The overlap included 55 CpG-SNP sites (Table S2). The most significant site (P = 4.40 × 10^−3^) in the CpG-SNP MWAS that overlapped with the GWAS data was located in the Roundabout, axon guidance receptor, homolog 2 gene (*ROBO2*). The overlapping CpG-SNPs included 26 genes present in GO. These genes were overrepresented (*P* < 0.01) in 12 level-5 GO terms (Table 3). The most significant term (P = 4.57 × 10^−4^) was “Regulation of synapse organization” which, among other genes, included *ROBO2*.

**Table 2:** Results from permutation-based enrichment tests of CpG-SNP MWAS and recent GWAS

GWAS	No. of mapped sites	No. of overlapping sites	Odds ratio	*P-*value
23andMe^a^	334,613	3	5.00	4.9 × 10^−3^
Converge	333,655	28	1.36	8.1 × 10^−2^
SSGAC	333,655	24	1.42	3.8 × 10^−2^

Strongest enrichment were detected using the top 1% threshold

^a^Due to limited access to the 23andMe GWAS data, instead of using thresholds for 1 and 5% the top 745 and 10,000 findings were used. Please see the Methods for details

**Table 3:** Over-represented gene ontology terms (*P* < 0.01) among genes detected from overlapping CpG-SNP MWAS and GWAS findings

Gene ontology (GO) term	Genes contained	*P-*value	*Q*-value
Regulation of synapse organization	3 (2.7%)	4.57 × 10^−4^	0.0292
Cell morphogenesis involved in differentiation	5 (0.8%)	1.59 × 10^−3^	0.0292
Regulation of cell morphogenesis	4 (0.9%)	2.94 × 10^−3^	0.0292
Positive regulation of nervous system development	4 (0.9%)	3.07 × 10^−3^	0.0292
Positive regulation of synapse assembly	2 (3.3%)	3.19 × 10^−3^	0.0292
Axon development	4 (0.9%)	3.42 × 10^−3^	0.0292
Axon guidance	3 (1.3%)	3.48 × 10^−3^	0.0292
Regulation of nervous system development	5 (0.7%)	3.54 × 10^−3^	0.0292
Central nervous system neuron development	2 (2.8%)	4.29 × 10^−3^	0.0315
Negative regulation of nervous system development	3 (1.1%)	5.81 × 10^−3^	0.0384
Regulation of cell projection organization	4 (0.7%)	7.44 × 10^−3^	0.0446
Regulation of extent of cell growth	2 (1.9%)	8.83 × 10^−3^	0.0486

“Genes contained” is the number of MWAS/GWAS implicated genes in each GO term with the percentage of all genes in the GO term in parentheses. All terms belong to the biological process category. Only level-5 terms were tested

## Discussion

Here we present the first MWAS of common CpG-SNPs (CpGs created/destroyed by SNPs with minor allele frequency > 10%) in MDD cases and controls. The methylation data were generated using a sequencing-based approach and involved 970,414 CpG-SNPs and 1132 individuals. Furthermore, we investigated the overlap of this study with recent GWAS for MDD, or related phenotypes. The MWAS suggested that multiple sites are potentially associated with MDD and resampling showed that the cumulative signal replicated. Furthermore, permutation-based enrichment tests suggested significant overlap with top findings from the MWAS and recent GWAS.

### Methylome-wide CpG-SNP analysis

The majority of the associated CpG-SNPs that expressed a significant meQTL effect and a significant dose effect in the MWAS showed a distinct pattern where methylation increased with the number of CpG alleles present, but where this increase was attenuated in MDD cases compared to controls. Thus, cases often showed less methylation than controls at the differently methylated loci. Many possible explanation may exist for this pattern. However, consistent with a general function of DNA methylation that protects the integrity of the genome by inactivating DNA elements^39,40^, this pattern would be in agreement with that a portion of potentially damaging mutations might not be properly silenced in MDD cases. Interestingly, the same pattern with less methylation observed in cases than in controls has previously been observed also in CpG-SNP studies for psychosis using both blood and brain tissue^31^.

### Overlap between CpG-SNP MWAS and GWAS

Many of the genes implicated by both the MWAS and the GWASs are of critical importance for neuronal function. Some of the overlapping gens have previously been associated with psychiatric disorders. For example, *ROBO2* (roundabout, axon guidance receptor, homolog 2) is critical for the maintenance of inhibitory synapses in the adult ventral tegmental area, a brain region important for the production of dopamine^41^, and has been implicated in schizophrenia^42–44^ and bipolar depression^45^. *ASIC2* (acid-sensing, proton-gated, ion channel 2) plays a role in neurotransmission^46^. *DCC* (deleted in colorectal carcinoma—netrin 1 receptor) upregulation in prefrontal cortex pyramidal neurons causes vulnerability to stress-induced social avoidance and anhedonia in mouse, and mutations in *DCC* have been associated with brain malformation^47^. Furthermore, *DCC* has been suggested to confer susceptibility to depression-like behaviors in mice and humans^48^ and was recently associated with mood instability, which has a strong genetic correlation to MDD^49^. In addition, the netrin 1 pathway, which involves *DCC*, has been identified as a candidate pathway for MDD^50^. Critically, both *ROBO2* and *DCC* interact in opposing fashion and have strong roles in directing axon pathfinding in developing neurons^51,52^. In summary, several of the genes detected in the MWAS-GWAS overlap serve critical biological functions of likely relevance to MDD etiology.

The overlap between the CpG-SNP MWAS and GWAS cannot be explained by the allele frequency differences between cases and controls that produce GWAS signals. It is true that methylation levels will be higher in the group with the higher frequency of the SNP allele that creates the CpG-SNP. However, these methylation differences are fully accounted for by the effect of the SNP as a “covariate” in the model we used for the CpG-SNP MWAS. Indeed, performing a GWAS with only the SNPs that were included in the CpG-SNP MWAS showed a lambda of 0.995 without any strong association signals (smallest *P*-value = 5.28 × 10^−6^). Thus, the CpG-SNP MWAS and GWAS provide additional and independent lines of evidence for the involvement of these loci in MDD.

## Conclusion

In the first CpG-SNP MWAS for MDD, we identified 27 suggestively significant sites. A significant number of these sites showed a negative CpG-SNP dose effect with less methylation in cases than controls. Furthermore, the MWAS results were over-represented among findings from three recent GWASs, which for example added additional support for the involvement of *DCC* in MDD. As the analysis approach prevents the methylation results to be driven by allele frequency differences between cases and controls, these results show that MWAS and GWAS provide additional and independent lines of evidence for the involvement of these loci in MDD. In conclusion, CpG-SNP methylation studies of MDD can contribute novel and biologically relevant information that complements previous findings detected by regular MWAS or GWAS alone.

### Availability of data and materials

Following local IRB approval individual level methylation data will be made available via dbGap (submission in preparation).

## Electronic supplementary material

Supplementary material
