# Supplementary material for: Convergence of evidence from a methylome-wide CpG-SNP association study and GWAS of major depressive disorder
Source: Transl Psychiatry. 2018 Aug 22;8:162. doi: 10.1038/s41398-018-0205-8 (PMC6105579; doi:10.1038/s41398-018-0205-8)
Supplement: Supplementary file 1 — Supplementary material [file 41398_2018_205_MOESM1_ESM.pdf]

Supplementary materials to:

Convergence of evidence from a methylome-wide CpG-SNP association study and GWAS of major depressive disorder

Supplementary tables, figure and notes 2

Table S1. Characteristics of participants........ 2
Genotype information 2
Quality control and CpG score calculation of methylation data 3
Figure S1. Regression plots of the 27 suggestively significant CpG-SNP MWAS associations. 6
Table S2. Sites included in overlap between CpG-SNP MWAS and GWAS 9
References........ 11

Supplementary tables and notes

Table S1. Characteristics of participants

	Controls		MDD		Test
	Mean	SD	Mean	SD	*P* value
	n = 320		*n = 812*		
Sex	0.591	0.492	0.667	0.471	0.018
Age	41.61	14.64	41.53	12.25	0.912
IDS	5.022	3.533	33.79	10.94	<0.001

Note: *n* is number of samples left after quality control. Sex indicates the proportion of males, age is measured in years. The IDS (Inventory of Depressive Symptomatology) is a self-report measure of symptom severity.

Genotype information

The NESDA participants were genotyped as previously described.[1] In short, the majority (95.2%) of DNA samples from the NESDA study were genotyped on Affymetrix 6.0 Human SNP array, while the remaining samples were genotyped on Perlegen-Affymetrix 5.0 array. In the quality control (QC) process, samples were excluded based on the following criteria: Affymetrix contrast QC < 0.4; missing rate > 10%; excess genome-wide heterozygosity or inbreeding levels (F < -0.075 or > 0.075); genotypes inconsistencies with reported gender; mendelian error rate > 5 standard deviations (SDs) from the mean of all samples; non-European/non-Dutch ancestry as indicated by principal component analysis.

SNPs were excluded for the following reasons: probes mapped badly against NCBI Build 37/UCSC hg19; minor allele frequency (MAF) < 0.005; missing rate > 5%; deviation from Hardy–Weinberg equilibrium (HWE) p< 1e-12;

SNPs present in both arrays were cross-imputed using GONL reference panel[2]. After imputation SNPs were converted to best guess genotypes using

Plink 1.90[3] and were removed if meeting the following more stringent criteria: a significant association with a single genotyping platform as compared to the other ($p < 10^{-5}$); an allele frequency difference > 10% with the GONL reference set; HWE $p < 10^{-5}$, Mendelian error rate > 5SDs (N>40); imputation quality $R^2 < 0.90$. The resulting data were then imputed using 1000G Phase 3 all ancestries reference panel via the Michigan Imputation Server[4]. Among the imputed SNPs, those retained for the present analyses met the following criteria: allele frequency difference < 5SDs of the mean of all SNPs with the reference set; HWE $p > 10^{-5}$, Mendelian error rate < 5 SDs; MAF >0.01; R^2>0.5.

Quality control and CpG score calculation of methylation data

As recently explained (Aberg et al submitted), we performed thorough quality control of reads, samples, and CpGs[5]. Of the 1,200 selected NESDA samples, 34 were excluded because the methylation enrichment (N=16) or library construction (*N*=18) failed. Reads aligning to loci without CpGs (non-CpGs) represent "noise" caused by, for example, alignment errors or imperfect enrichment leading to non-methylated fragments being sequenced. The average non-CpG to CpG coverage ratio was 0.012 (SD=0.025). Using a threshold of 0.05 to remove samples with high "noise" levels (N=10), left an average non-CpG to CpG coverage ratio of 0.010 (SD=0.005) in the remaining samples. For 10 samples, sequence variants called from the methylation data did not match the genotype information obtained from a previous GWAS of these samples[6]. This indicated that a sample swap or sample contamination may have occurred. As it was not possible to determine whether the problem occurred in the GWAS or

MWAS data, we conservatively excluded all 10 samples from further analysis. Finally, we used the R function 'pcout' in the 'mvoutliers' package (with the upper boundary for outlier detection set to 15, the scaling constant set to 0.5, and the boundary for final outliers set to 0.2) to identify multidimensional outliers using the first 15 principal components of the methylation data as input. Fourteen samples were identified as multidimensional outliers and omitted. This left a sample of 1,132 subjects for statistical analysis.

The mean number of reads for the 1,132 remaining samples was 59.4 million (SD=11.2 million) of which, on average, 99.1% aligned. Aligned reads were subjected to further quality control. Although reads often map to multiple genomic locations, in most cases, a single alignment can be selected because it is clearly better than other alignments. In the case of multi-reads, multiple alignments receive equally good alignment scores. When Bowtie2 encounters multi-reads, it uses a pseudo-random number generator to select a single primary alignment. Duplicate-reads are reads that start at the same nucleotide positions. When sequencing a whole genome, duplicate-reads typically arise from artifacts in template preparation or amplification. However, in the context of sequencing an enriched genomic fraction, duplicate-reads are increasingly likely to occur because reads originate from a smaller fraction of the genome. Therefore, only when more than 3 (duplicate) reads start at the same position, we reset the read count to 1 implicitly assuming these reads are tagging a single clonal fragment. This left an average of 48.7 million reads per sample (=81.9% of all reads).

To identify all common CpGs, we combined reference genome sequence (hg19/GRCh37) with SNP information from the European super-population on the 1000 Genomes project (Phase 3). To avoid analyzing sites that are CpGs in only a very small proportion of subjects, we excluded CpGs created/destroyed by SNPs that had a minor allele frequency <1%. This resulted in 27,916,990 CpGs. Additionally, CpGs in loci prone to alignment errors, e.g., in repetitive regions, were eliminated prior to the analysis. To identify these CpGs, we used RaMWAS to perform an *in silico* alignment experiment outlined elsewhere that aligns all possible reads to the reference[5]. The vast majority of CpGs (89.3%) were located in regions that showed perfect alignment coverage. Only 1.3% of the CpGs showed evidence of alignment problems (defined as 15% or more reads from this locus not aligning properly) and were removed from further analyses. Finally, akin to filtering SNPs with low minor allele frequency, we eliminated 5,682,206 CpGs with average coverage less than 0.3. These sites may create false positive MWAS findings due to low power or statistical problems associated with analyzing sparse data. After all quality control, 21,869,561 CpGs remained. Among these, 970,414 were CpGs that can be created or destroyed by common SNPs available for methylome-wide association study (MWAS) of CpG-SNPs.

Methylation scores were calculated by estimating the number of fragments covering the CpG using a non-parametric estimate of the fragment size distribution[7]. These scores provide a relative measure of the amount of methylation for each individual at that specific site.

Figure S1. Regression plots of the 27 suggestively significant CpG-SNP MWAS associations.

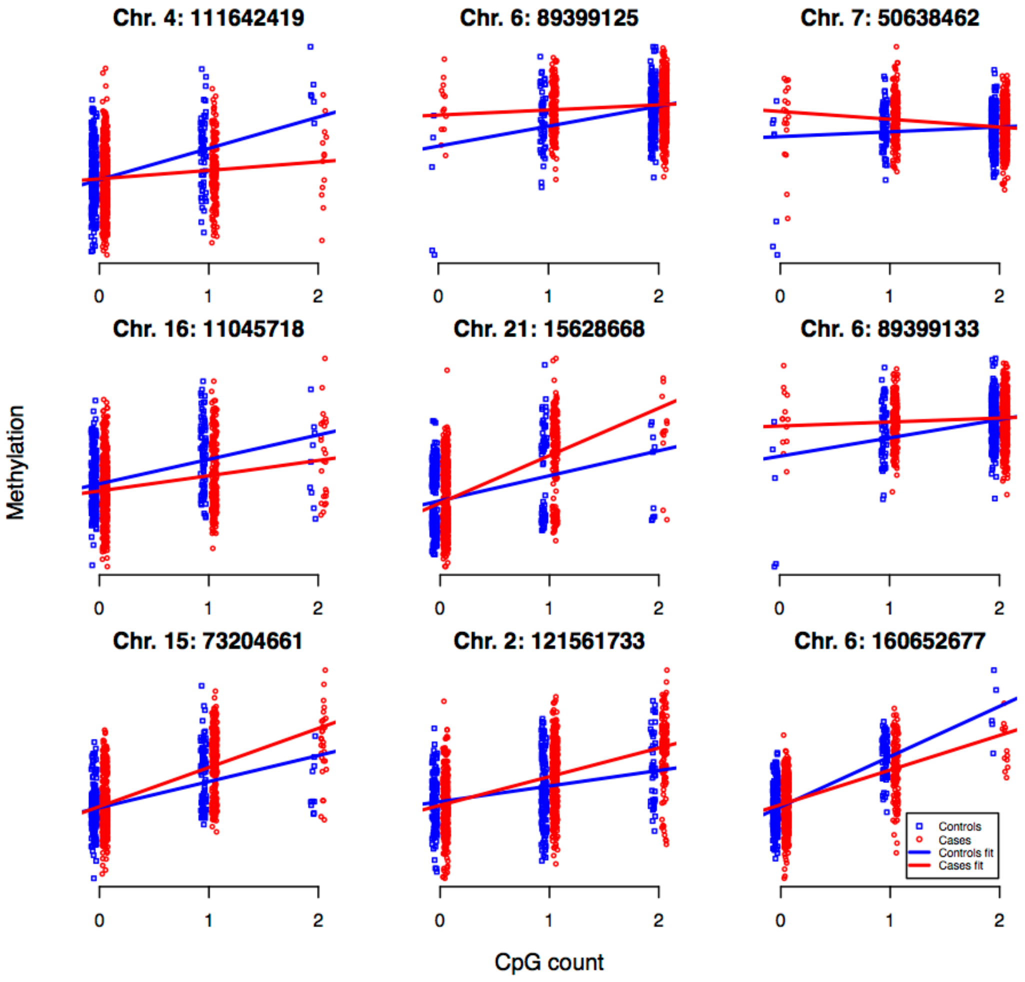

The methylation coverage (y-axis) is plotted for cases (red circles) and controls (blue squares) against the CpG count (x-axis). The fitted regression lines are indicated for cases (red) and controls (blue).

Figure S1 - continued. Regression plots of the 27 suggestively significant CpG-SNP MWAS associations.

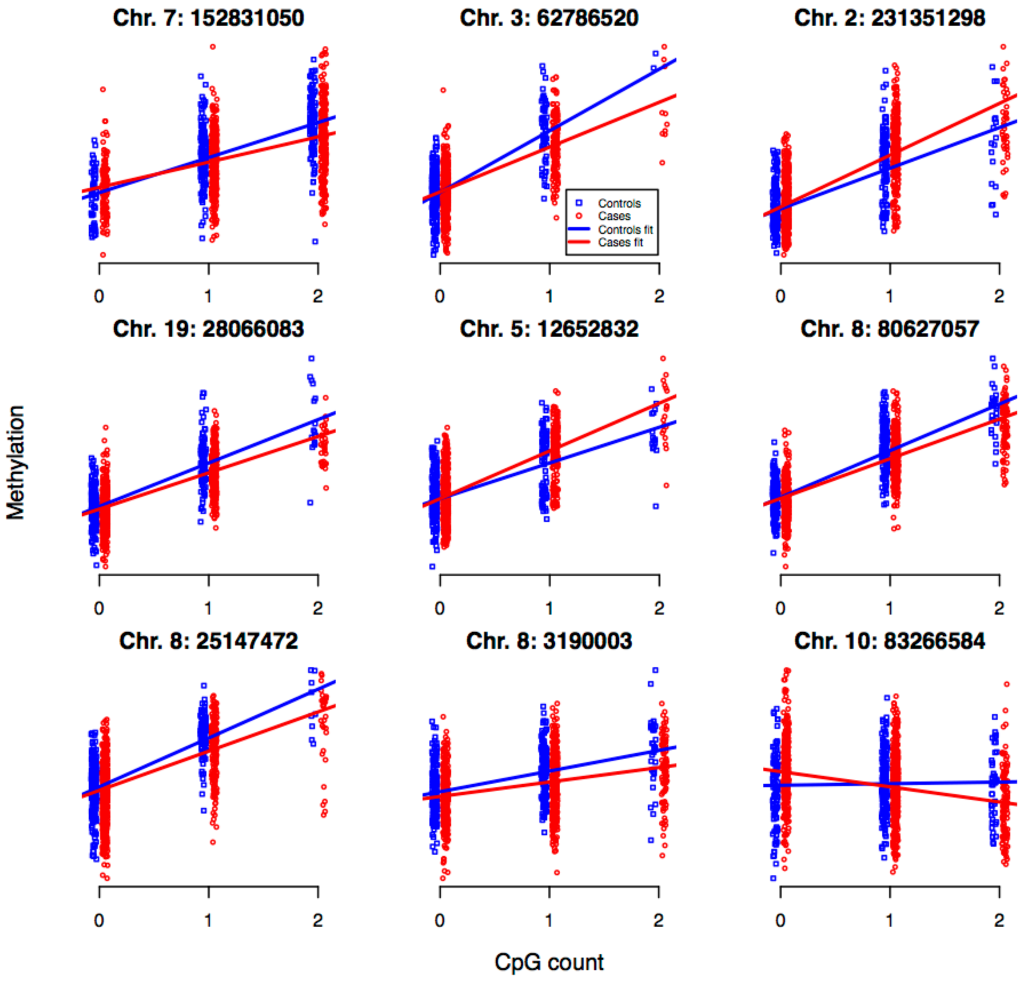

The methylation coverage (y-axis) is plotted for cases (red circles) and controls (blue squares) against the CpG count (x-axis). The fitted regression lines are indicated for cases (red) and controls (blue).

Figure S1 - continued. Regression plots of the 27 suggestively significant CpG-SNP MWAS associations.

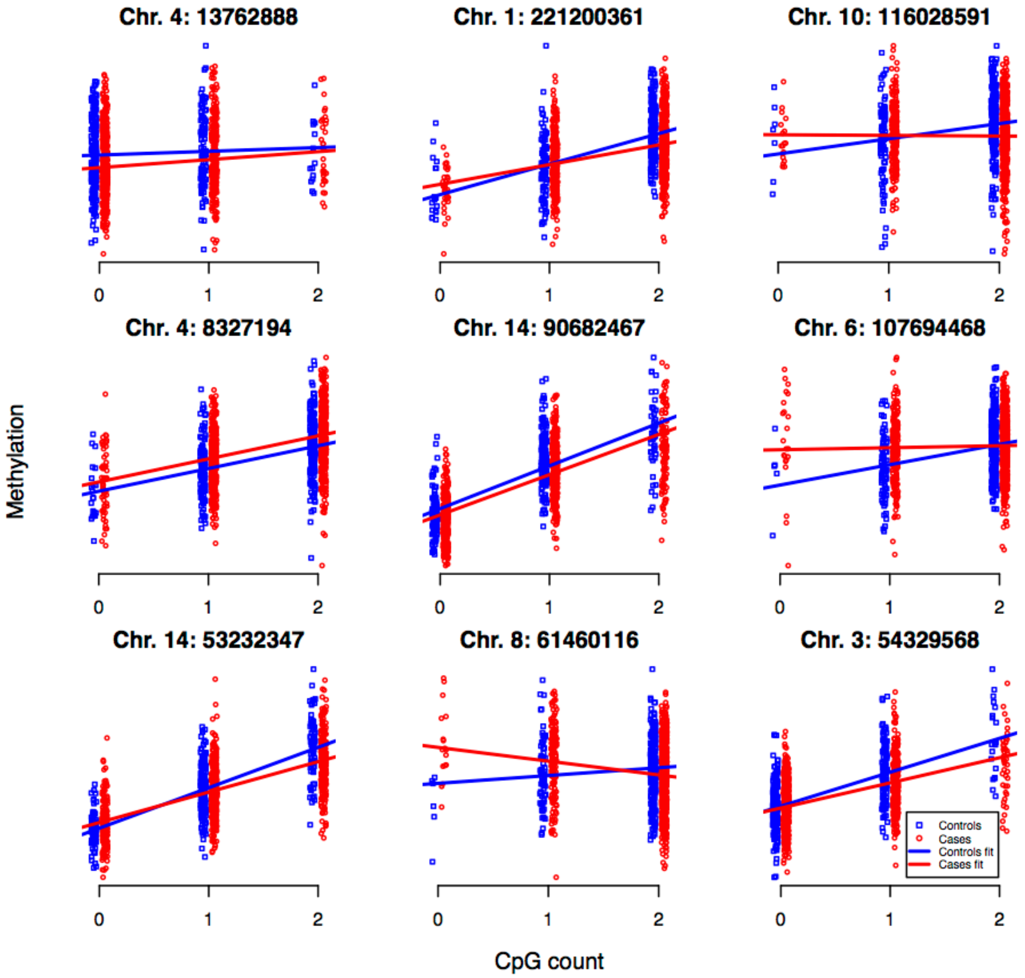

The methylation coverage (y-axis) is plotted for cases (red circles) and controls (blue squares) against the CpG count (x-axis). The fitted regression lines are indicated for cases (red) and controls (blue).

Table S2. Sites included in overlap between CpG-SNP MWAS and GWAS

Chr.	CpG start	CpG end	CpG-SNP MWAS Corrected *P*	hgnc symbol	Rs number	GWAS
2	53,682,501	53,682,502	5.31E-03		rs4672433	Converge
2	153,102,914	153,102,915	7.95E-03		rs11679305	Converge
2	161,916,845	161,916,846	2.46E-03		rs1114339	SSGAC
2	228,860,909	228,860,910	3.79E-03	SPHKAP	rs11883583	Converge
2	228,887,673	228,887,674	6.35E-03	SPHKAP	rs6736430	Converge
2	236,766,646	236,766,647	3.79E-03	AGAP1	rs10208161	Converge
3	13,106,302	13,106,303	7.93E-03	IQSEC1	rs360875	Converge
3	34,826,776	34,826,777	8.88E-04		rs622563	SSGAC
3	49,690,496	49,690,497	2.50E-03	BSN	rs11709525	SSGAC
3	71,983,098	71,983,099	2.07E-03		rs11706411	SSGAC
3	76,566,129	76,566,130	5.25E-05	ROBO2	rs3901063	Converge
4	6,344,983	6,344,984	3.43E-03	PPP2R2C	rs4689413	SSGAC
4	9,997,112	9,997,113	3.23E-03	SLC2A9	rs4529048	SSGAC
4	104,233,989	104,233,990	5.00E-03		rs11938459	Converge
4	111,278,757	111,278,758	2.50E-03		rs472305	Converge
4	138,963,965	138,963,966	2.74E-04	LINC00616	rs4863756	SSGAC
5	39,908,712	39,908,713	8.84E-04		rs582489	SSGAC
5	45,716,209	45,716,210	3.86E-04		rs2879074	SSGAC
5	87,513,775	87,513,776	7.93E-03	TMEM161B	rs357513	23andMe
5	87,592,024	87,592,025	5.65E-03	TMEM161B-AS1	rs247909	23andMe
5	164,413,592	164,413,593	2.59E-03		rs10057578	SSGAC
6	31,107,733	31,107,734	6.93E-03	PSORS1C1	rs1966	Converge
6	35,114,542	35,114,543	1.29E-03	TCP11	rs2038740	Converge
6	134,582,426	134,582,427	3.21E-03	SGK1	rs4896036	Converge
6	163,612,783	163,612,784	1.55E-03	PACRG	rs6937392	Converge
7	125,774,883	125,774,884	6.66E-03		rs607038	Converge
7	133,345,526	133,345,527	4.44E-03	EXOC4	rs7792396	SSGAC
8	11,646,934	11,646,935	4.10E-03		rs904015	SSGAC
9	11,602,902	11,602,903	2.03E-03		rs4524892	SSGAC
9	120,510,180	120,510,181	3.09E-03		rs10818080	SSGAC
10	133,253,402	133,253,403	6.71E-03		rs3123187	SSGAC
11	87,134,768	87,134,769	7.23E-04		rs1001592	Converge
12	14,846,187	14,846,188	7.54E-03	GUCY2C	rs11056101	Converge
13	29,096,237	29,096,238	1.47E-03		rs9506000	Converge
13	53,858,586	53,858,587	2.20E-03		rs9596774	23andMe
13	94,051,114	94,051,115	7.40E-03	GPC6	rs2762111	SSGAC

14	72,977,524	72,977,525	7.19E-03	RGS6	rs2286069	SSGAC
14	95,337,640	95,337,641	5.54E-03		rs8006342	Converge
14	104,559,919	104,559,920	7.44E-03	ASPG	rs1770984	Converge
15	88,456,089	88,456,090	7.77E-04	NTRK3	rs3903308	Converge
16	5,061,971	5,061,972	8.39E-03	SEC14L5	rs1558560	Converge
16	23,563,501	23,563,502	2.02E-03	EARS2	rs7187920	Converge
16	52,638,503	52,638,504	4.42E-03	CASC16	rs3104788	SSGAC
16	87,988,440	87,988,441	3.10E-04	BANP	rs7194067	Converge
17	31,798,177	31,798,178	3.91E-03	ASIC2	rs1553496	Converge
17	43,513,441	43,513,442	9.35E-04	PLEKHM1	rs11012	SSGAC
17	77,889,833	77,889,834	6.25E-03		rs1696784	Converge
18	8,363,474	8,363,475	7.33E-03	PTPRM	rs1318213	Converge
18	22,989,031	22,989,032	5.65E-03		rs7240037	Converge
18	50,608,436	50,608,437	5.09E-03	DCC	rs9949889	SSGAC
18	57,136,111	57,136,112	3.78E-03	CCBE1	rs17065849	Converge
18	57,837,224	57,837,225	3.88E-03		rs1619975	SSGAC
18	69,361,739	69,361,740	5.31E-03		rs1942355	SSGAC
19	46,289,503	46,289,504	2.27E-03	DMWD	rs8109951	SSGAC
22	22,036,512	22,036,513	4.50E-03	PPIL2	rs881091	SSGAC

Note: Chr. is chromosome. * Corrected P values are reported.

References

1. Mbarek H, Milaneschi Y, Hottenga JJ, Ligthart L, de Geus EJC, Ehli EA *et al.* Genome-Wide Significance for PCLO as a Gene for Major Depressive Disorder. *Twin Res Hum Genet* 2017; **20**(4)**:** 267-270.

2. Fedko IO, Hottenga JJ, Medina-Gomez C, Pappa I, van Beijsterveldt CE, Ehli EA *et al.* Estimation of Genetic Relationships Between Individuals Across Cohorts and Platforms: Application to Childhood Height. *Behav Genet* 2015; **45**(5)**:** 514-528.

3. Chang CC, Chow CC, Tellier LC, Vattikuti S, Purcell SM, Lee JJ. Second-generation PLINK: rising to the challenge of larger and richer datasets. *Gigascience* 2015; **4:** 7.

4. Das S, Forer L, Schonherr S, Sidore C, Locke AE, Kwong A *et al.* Next-generation genotype imputation service and methods. *Nat Genet* 2016; **48**(10)**:** 1284-1287.

5. Aberg KA, McClay JL, Nerella S, Xie LY, Clark SL, Hudson AD *et al.* MBD-seq as a cost-effective approach for methylome-wide association studies: demonstration in 1500 case--control samples. *Epigenomics* 2012; **4**(6)**:** 605-621.

6. Sullivan PF, de Geus EJ, Willemsen G, James MR, Smit JH, Zandbelt T *et al.* Genome-wide association for major depressive disorder: a possible role for the presynaptic protein piccolo. *Mol Psychiatry* 2009; **14**(4)**:** 359-375.

7. van den Oord EJ, Bukszar J, Rudolf G, Nerella S, McClay JL, Xie LY *et al.* Estimation of CpG coverage in whole methylome next-generation sequencing studies. *BMC Bioinformatics* 2013; **14**(1)**:** 50.

8. Desmet F-O, Hamroun D, Lalande M, Collod-Béroud G, Claustres M, Béroud C. Human Splicing Finder: an online bioinformatics tool to predict splicing signals. *Nucleic Acids Research* 2009; **37**(9)**:** e67-e67.
